# Gaming Instead of Training? Exergaming Induces High-Intensity Exercise Stimulus and Reduces Cardiovascular Reactivity to Cold Pressor Test

**DOI:** 10.3389/fcvm.2022.798149

**Published:** 2022-01-27

**Authors:** Sascha Ketelhut, Reinhard G. Ketelhut, Eva Kircher, Lisa Röglin, Kuno Hottenrott, Anna Lisa Martin-Niedecken, Kerstin Ketelhut

**Affiliations:** ^1^Institute of Sport Science, Martin Luther University Halle-Wittenberg, Halle (Saale), Germany; ^2^Medical Center Berlin (MCB), Cardiology and Sports Medicine, Berlin, Germany; ^3^Charité University Medicine Berlin, Virchow-Klinikum, Berlin, Germany; ^4^Department of Design, Institute for Design Research, Zurich University of the Arts, Zurich, Switzerland; ^5^Faculty of Natural Science, MSB Medical School Berlin, Berlin, Germany

**Keywords:** exergaming, cold pressor test, high-intensity exercise, games for health, blood pressure reactivity, hemodynamics, serious games, pulse wave velocity

## Abstract

**Introduction:**

The present study assessed if an exercise session in an innovative exergame can modulate hemodynamic reactivity to a cold pressor test (CPT) to a similar extent as a typical moderate endurance training (ET). Furthermore, cardiorespiratory, and affective responses of an exergame session and an ET were compared.

**Methods:**

Twenty-seven healthy participants aged 25 ± 4 years (48% female; BMI 23.0 ± 2.1 kg/m^2^) participated in this cross-sectional study. All participants completed both an ET on a treadmill and training in the ExerCube (ECT). HR and oxygen consumption were recorded during both training sessions. Before and after both exercise sessions, the hemodynamic reactivity to a CPT was determined.

**Results:**

During ECT, HR, oxygen consumption, energy expenditure, and the metabolic equivalent of the task were significantly higher than those obtained during ET (*p* < 0.001). With regard to the CPT, the participants showed significantly lower responses in peripheral systolic (*p* = 0.004) and diastolic blood pressure (*p* = 0.009) as well as central systolic (*p* = 0.002) and diastolic BP (*P* = 0.01) after ECT compared to ET. The same was true for pulse wave velocity (*p* = 0.039).

**Conclusion:**

The ECT induced a significantly higher exercise stimulus compared to the ET. At the same time, it attenuated hemodynamic stress reactivity. The ECT presents a relevant training stimulus that modulates cardiovascular reactivity to stress, which has been proven as a predictor for the development of hypertension.

**Trial Registration:**

ISRCTN registry, ISRCTN43067716, 14 April 2020, Trial number: 38154.

## Introduction

Exergames are interactive video games that require the user to move their body in order to progress through the game. Already a decade ago, health professionals and scholars have identified exergames as a promising approach to facilitate physical activity (1). Due to its attractive and immersive design, exergames can reach people who are not so prone to engaging in physical activity. According to the literature, exergames can increase physical activity adherence (2), as players find virtual versions of traditional exercises more enjoyable (3). Even though enjoyment is an important component for engaging in physical activity (4), an exercise stimulus not only has to be fun but should also reach a certain threshold to induce physiological adaptions and thus relevant health effects.

Even though, numerous studies have determined a higher exercise intensity in exergames compared to normal sedentary videogames, the literature remains equivocal as to whether exergames elicit intensity levels that conform to physical activity guidelines (5). Accordingly, relevant physiological responses and health benefits are questionable. However, the new generations of exergames not only aim for an exciting and appealing gaming experience but started to integrate sound training programs to achieve more health benefits (6, 7). With the ExerCube, a promising exergame came on the market that combines an attractive game design and an adaptive functional training stimulus (8, 9). The ExerCube implements an innovative game design that continuously adapts the difficulty and complexity of the game according to the player's cognitive skills and fitness level. Thus, it is one of the first games that may allow an individually tailored training stimulus.

In previous studies evaluating different exergames, the focus was on assessing affective and respiratory responses. Surprisingly, very few studies have assessed the effects on direct health outcomes such as blood pressure (BP) and other hemodynamic parameters. Studies that have examined the short- and long-term effects of exergaming on BP show inconsistent results (2, 10, 11).

To the best of our knowledge, no studies have assessed the effects of exergaming on hemodynamic reactivity to a standardized stress test. Cardiovascular hyperreactivity to stress has been identified as an important risk factor for developing future hypertension and cardiovascular diseases (12). The cold pressor test is a common and extensively validated test for assessing cardiovascular reactivity (13). Previous studies show that better physical fitness and higher levels of physical activity correspond with a lower cardiovascular response to the cold pressor test (14). Furthermore, it has been demonstrated that acute exercise can positively modulate hemodynamic responses during a cold pressor test (15–17). It remains questionable if exergaming can have similar effects on hemodynamic reactivity.

The present study is the first to determine whether an exergaming session in a new and innovative exergame called the ExerCube triggers relevant cardiorespiratory responses like a typical moderate endurance training (ET). Furthermore, it assesses if an exergaming session may also modulate acute hemodynamic reactivity to a cold pressor test. The results help determine if this exergame may be an attractive and effective training tool for the prevention of cardiovascular diseases.

## Materials and Methods

### Participants and Study Design

The participants were recruited from the student body of the Martin-Luther-University Halle-Wittenberg. Exclusion criteria included an underlying health condition, orthopedic injuries, regular use of antihypertensive or other cardiovascular medications. The inclusion criteria for female participants further required a regular and healthy menstrual cycle for the past 6 months or more. A total of 27 recreationally active participants (aged 25 ± 4 years; body mass index 23.2 ± 2.3 kg/m^2^; 48 % female) volunteered to participate in the study. All received a verbal and written explanation of the study's objective, experimental procedures, and the risks and benefits associated with the study. Each participant than gave a written informed consent. The experimental procedures of this cross-sectional study were approved by the institutional research committee (Medical Faculty of the Martin-Luther-University Halle-Wittenberg 2019-177). The participants took part in three experimental sessions. For each session, they were asked to visit the laboratory at least 4 hours postprandial, and to refrain from consuming caffeinated or alcoholic beverages and nicotine for 4 hours. They were further advised to abstain from intensive physical exercise for at least 12 hours (18).

The experimental sessions were held at least 48 h apart and occurred at approximately the same time of day. All tests were performed under standardized conditions in the same laboratory with a room temperature of 23.3 ± 0.5°C. For female participants, the examination days were selected so that they did not fall into the early follicular phase.

On the first day, demographic and anthropometric data were obtained. Furthermore, resting energy expenditure was assessed, and a graded exercise test was conducted. On the second and third visit, participants completed an ET on a treadmill and a training in the ExerCube (ECT) in a random and counterbalanced order. The order was randomized using the computer program random sequence generator at www.random.org.

### Test Procedure

#### Baseline Examination

Participants completed baseline questionnaires that assessed the demographics, habitual physical activity, and medical history. Height and body mass were measured with a stadiometer and a scale (BC-545 Innerscan, Tanita, Netherlands). The waist circumference was measured to the nearest 0.1 cm midway between the lowest ribs and the iliac crest.

Resting energy expenditure was assessed *via* indirect calorimetry (MetaMax 3B Cortex, Leipzig, Germany). The participants rested in a supine position in a quiet, darkened room emotionally undisturbed. After 10 min of resting, values were recorded. Energy expenditure was measured for ≈15 min with the person calm but awake. Resting energy expenditure was obtained when the participants attained a steady state. Steady states were defined as time intervals of at least 5 min, in which every average minute oxygen consumption and carbon dioxide production changed by <10%, and the average respiratory quotient changed by <5%. The energy expenditure was calculated from the VO_2_ and VCO_2_ using the Weir equation (19).

The participants then completed a graded exercise test on a treadmill (h/p/cosmos sports & medical GmbH, Pulsar 4.0, Nussdorf-Traunstein, Germany) to voluntary exhaustion. The initial speed was set between 7.5 and 10.5 km/h. Each step lasted 3 min, interspersed with a 1 min passive rest to draw lactate samples from the earlobe. After each step, the speed was increased by 1.5 km/h until volitional exhaustion.

#### ExerCube Training

The ExerCube (Sphery AG, Au, Switzerland) is an adaptive fitness game setup (20) that allows the player to engage in an individually tailored, whole-body, functional exercise session. The game setup consists of three walls that surround the player. The game scenario is projected onto all three walls allowing an immersive gaming experience. The player wears two HTC Vive-trackers on their wrists and two on their ankles which continuously track the player's movements and body position. The participants played the game Sphery Racer (9, 20). During the game the player navigates an avatar along a virtual racetrack and must perform various movement tasks. The motion capturing system analyzes the timing and accuracy of movements throughout the game which guarantees a correct execution of the various movement tasks. The game scenario in the present study lasted about 27 min and implemented six exercise levels, which gradually increased in intensity. The levels are interspersed by short (≈30 s) rest periods. While playing, the game continuously adapts the difficulty and complexity according to the player's cognitive skills. If the player makes too many mistakes, the speed, and complexity of the game decrease. If the player makes no mistakes, both parameters increase gradually. Furthermore, the game allows setting an individual heart rate (HR) threshold. Whenever the player reaches a predetermined HR during the game, the speed of the game slows down. Thus, this game allows an individually tailored training stimulus.

Ventilation and HR were continuously assessed throughout the exercise session. Before and after exercise, body mass was assessed. Additionally, the participants were asked to fill out a questionnaire assessing intrinsic motivation.

#### Moderate Endurance Exercise

The ET consisted of a 35 min moderate endurance exercise on a treadmill. After a 5 min warm-up (5.5 km/h), treadmill speed was adjusted according to the individual aerobic threshold. The speed was then continuously adjusted so that the participants reached a HR of ≈65% of their maximum HR (HRmax). Ventilation and HR were continuously assessed throughout the exercise session. Before and after the exercise session, body mass was assessed. Immediately after the end of the game, the participants were asked to complete the “short scale on intrinsic motivation.”

#### Cold Pressor Test

The cold pressor test is a standardized test widely used to assess the hemodynamic reactivity to a stressor. Cardiovascular hyperreactivity to stress has been identified as a mechanism in the pathogenesis of cardiovascular diseases and is a marker reflecting cardiovascular risk (12).

The cold pressor test was conducted by trained study staff before and 45 min after each exercise session using a standardized protocol. Prior to the cold pressor test, rest measurements were carried out in supine position. Subsequently, the participants immersed their right hand (fingers spread) up to the wrist into cold water (5.0°C) for 2 min. Both during rest as well as during cold-water exposure peripheral systolic (pSBP) and diastolic (pDBP) pressure, central systolic (cSBP) and diastolic (cDBP) pressures, pulse wave velocity, and HR were determined on the left upper arm with the Mobil-O-Graph (IEM, Stolberg, Germany) as a clinically validated device for hemodynamic measurements (21). Hemodynamic reactivity was determined as the changes (Δ) in the respective parameters between the rest measurement and the subsequent cold pressor test. After completing the test, participants rated the pain intensity and discomfort on a verbal descriptor scale.

### Experimental Measures

#### Heart Rate and Blood Lactate

HR was monitored throughout the graded exercise test, ECT, and ET with a Polar heart rate monitor (Polar Electro OY, Kempele, Finland) using a chest strap. Blood lactate concentration was assessed after each stage of the graded exercise test by enzymatic amperometry (Dr. Müeller Gerätebau GmbH, Super GL ambulance, Freital, Germany). Small blood samples were drawn from the earlobe with a lancet. The collected data were processed utilizing the software WinLactat 3.1 (mesics GmbH, Münster, Germany), and individual thresholds were derived from the lactate-velocity curve using the Dickhuth model (22).

#### Ventilation

VO_2_ and CO_2_ were recorded continuously (breath-by-breath) during the graded exercise test, the ECT, and the ET. A portable indirect calorimetric gas-exchange analysis system MetaMax 3B (Cortex Biophysik GmbH, Leipzig, Germany) was used to allow unrestricted movement. The values obtained were averaged over 30-s epochs. Prior to each testing session, a two-point calibration procedure was conducted according to the manufacturer's guidelines. The calibration of the oxygen and carbon dioxide sensors was performed with gases of known concentrations. The flow rate was calibrated with a 3-liter volume syringe, according to the manufacturer's instructions. Ambient air measurements were conducted before each test.

#### Enjoyment/Intrinsic Motivation

A modified German version of the Intrinsic Motivation Inventory (23) was used to assess intrinsic motivation. The questionnaire short scale on intrinsic motivation (Kurzskala intrinsischer Motivation) (24) contains 12 of the original 45 items. It assesses the participants' interest/enjoyment, the perceived competence, the perceived choice, and perceived pressure and tension while performing a particular activity. The scale consists of four subscales with three items each. Due to the study design the subscale perceived choice was excluded. The questionnaire represents an objective, reliable, valid (internal consistency between 0.89 and 0.79), and time-efficient tool for measuring intrinsic motivation (24).

### Statistics

An a priori power analysis was conducted utilizing G^*^power (Version 3.1.2; Heinrich Heine Universität, Dusseldorf, Germany). Assuming an effect size of 0.8 with an alpha level 0.05 using a group effect on change in pSBP as the primary outcome measure, 24 participants are required. Accounting for possible dropouts 27 participants were recruited.

All statistical analysis were performed using IBM SPSS Statistics v. 27.0 (SPSS, Chicago, IL, USA). The results are presented as means ± standard deviation. Individual HRmax and maximum oxygen consumption (VO_2_max), measured during the graded exercise test, were used to calculate the percentage of HRmax and VO_2_max achieved during the exercise sessions for each participant. The individual metabolic equivalent of task (MET) was calculated by dividing the respective activity VO_2_ by resting VO_2_. Age-specific MET cut-points were used to interpret the intensity of activities: light (2.4–4.7 METs), moderate (4.8–7.1 METs), and vigorous (>7.2 METs) (26).

Paired samples *t*-tests were used to compare the outcome measures during the ECT, and the ET. To compare the changes in hemodynamic parameters and perceived pain during the cold pressor tests a 2 x 2 ANOVA with repeated measures was used to test main effect and interactions for time (pre or post) and exercise (ECT or ET). Sex was included as a covariate. The Levene test was used to check the homogeneity of variance.

## Results

All participants completed the three examination sessions with no adverse events. The characteristics of the participants are presented in Table 1. The peak responses to the graded exercise test can be found in Table 2. According to the body mass index, four participants were classified as overweight (>25). Based on the waist-to-height ratio, two of the participants included showed values in the overweight range. According to pSBP, none of the participants could be classified as hypertensive (25). Two of the participants presented a high normal pSBP. According to the pDBP, all participants were classified as normotensive.

**Table 1:** Participants' characteristics.

**Outcomes**	**Mean value and Standard deviation**	**Range**
Age (years)	25 ± 3.8	18–35
Gender (f/m)	13/14	
Body mass (kg)	68.5 ± 10.7	47.3–86.5
Height (cm)	172 ± 9.7	153–186
Body-Mass-Index (kg/m^2^)	23.0 ± 2.1	19.9–27.6
Waist-to-Height-Ratio	0.43 ± 0.1	0.38–0.52
pSBP (mmHg)	120 ± 8.3	106–138
pDBP (mmHg)	70 ± 6.2	60–84
REE (kcal/min)	1.2 ± 0.2	0.9–1.6

*pSBP, resting peripheral systolic blood pressure; pDBP, resting peripheral diastolic blood pressure; REE, resting energy expenditure*.

**Table 2:** Peak responses to the graded treadmill exercise test.

**Outcomes**	**Mean value and standard deviation**	**Range**
VO_2_max (ml/kg/min)	49.4 ± 5.9	39–62
VO_2_max (L/min)	3.40 ± 0.76	2.18–4.84
HRmax (bpm)	194 ± 8	176–207
Lactate (mmol)	8.8 ± 2.0	4.5–12.5

*VO_2_max, maximal oxygen consumption during the graded exercise test; HRmax, maximal heart rate during the graded exercise test*.

**Figure 1:**
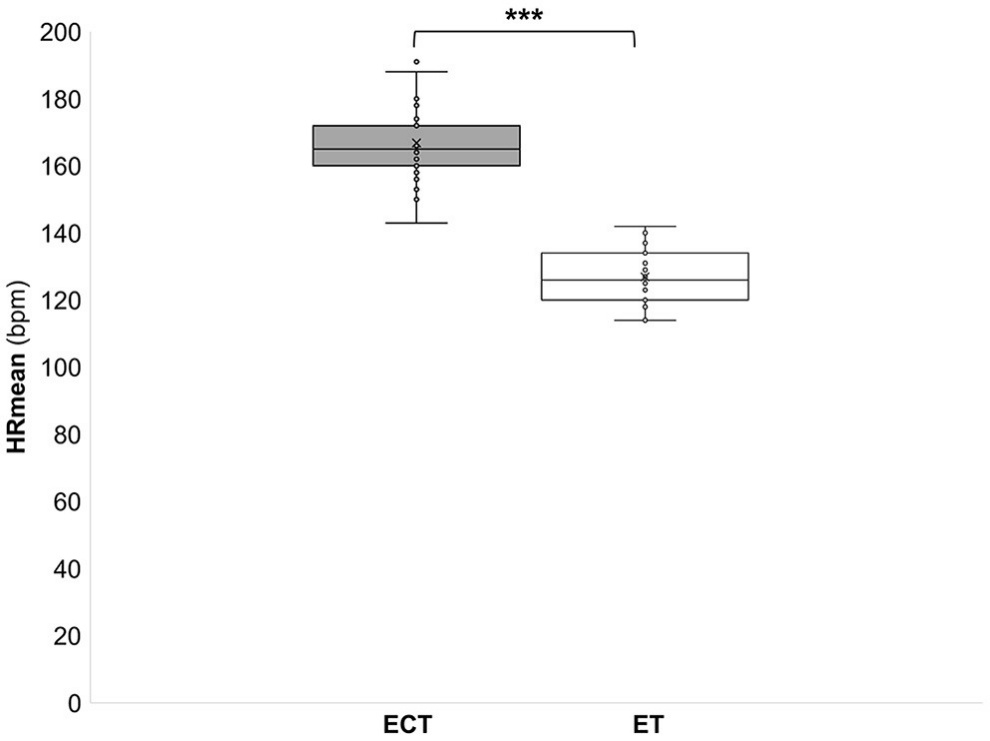
Mean heart rate (HRmean) during a training in the ExerCube **(ECT)** and a moderate endurance training **(ET)**. ***p < 0.001.

The participants achieved a peak HR (HRpeak) of 187 ± 9 bpm during the ECT, which corresponds to 97 ± 4% of their individual HRmax. Throughout the game, the mean HR (HRmean) was 167 ± 11 bpm, which corresponds to 86 ± 4% of HRmax (Figure 1).

**Figure 2:**
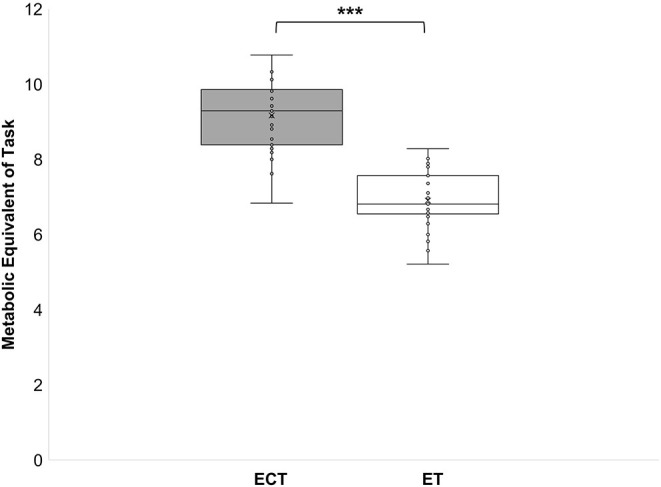
Mean oxygen consumption (VO_2_mean) during a training in the ExerCube **(ECT)**, and a moderate endurance training **(ET)**. ***p < 0.001.

**Figure 3:**
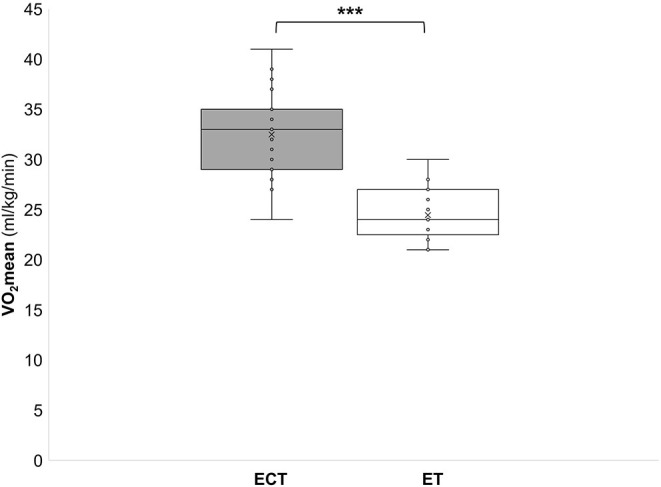
Metabolic equivalent of task during a training in the ExerCube **(ECT)**, and a moderate endurance training **(ET)**. ***p < 0.001.

**Figure 4:**
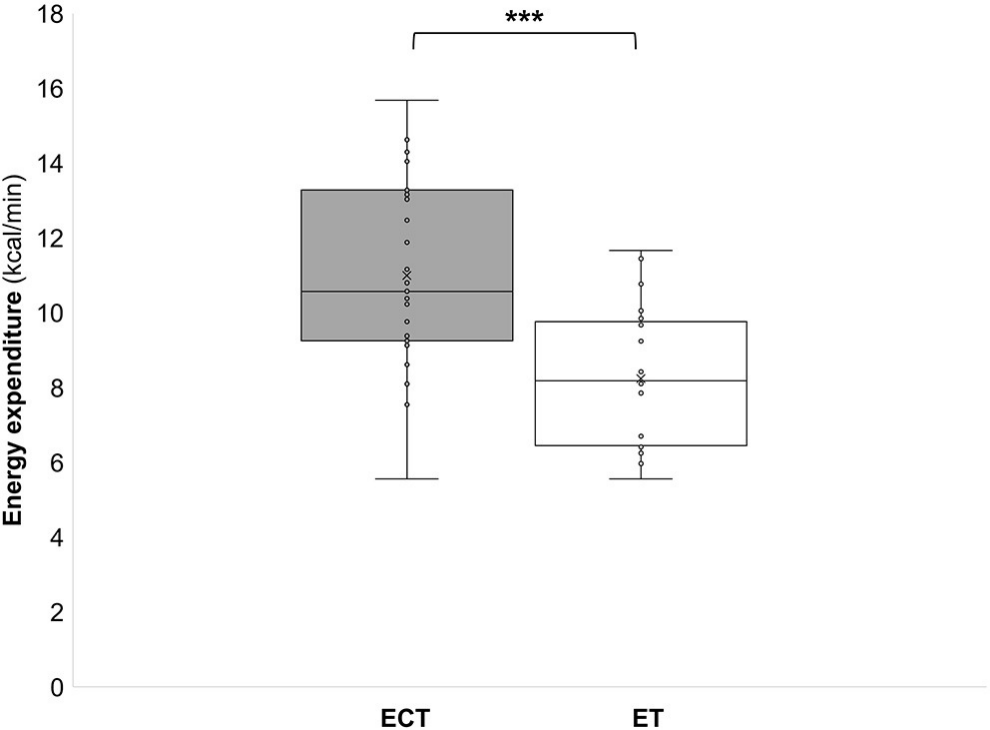
Energy expenditure during a training in the ExerCube **(ECT)**, and a moderate endurance training **(ET)**. ***p < 0.001.

**Figure 5:**
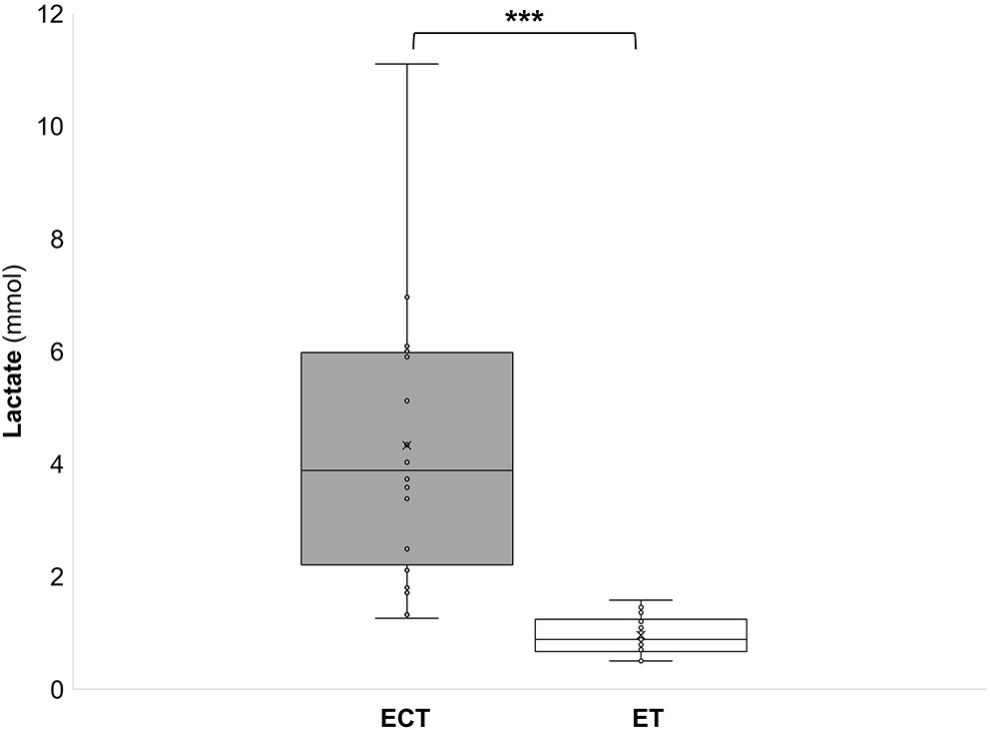
Lactate values during a training in the ExerCube **(ECT)**, and a moderate endurance training **(ET)**. ***p < 0.001.

During the ECT the peak oxygen consumption (VO_2_peak) reached 41.6 ± 5.2 ml/kg/min, which corresponds to 84 ± 8% of the maximum oxygen consumption (VO_2_max). The mean oxygen consumption (VO_2_mean) reached 32.5 ± 4.1 ml/kg/min which corresponds to 66 ± 6% of VO_2_max (Figure 2). During the ET HRpeak (151 ± 10 bpm; 78 ± 4% of HRmax), HRmean [127 ± 8 bpm; 6 ± 4% of HRmax (Figure 1)], VO_2_peak (31.4 ± 3.6 ml/kg/min; 64 ± 6 % VO_2_max), and VO_2_mean [24.5 ± 2.6 ml/kg/min; 48 ± 11% of VO_2_max (Figure 2)] were significantly (*p* < 0.001) lower. Furthermore, MET (6.7 ± 1.6 vs. 9.2 ± 0.9), energy expenditure (7.9 ± 2.4 vs. 10.9 ± 2.6 kcal/min), and lactate values (4.3 ± 2.4 vs. 0.9 ± 0.4 mmol) were significantly (*p* < 0.001) lower during the ET compared to the ECT (Figures 3–5).

The intrinsic motivation score was significantly (37.6 ± 3.9, *p* < 0.001) higher during the ECT compared to ET (32.1 ± 5.4). Especially the subscale score for interest and enjoyment was significantly higher during the ECT (Table 3).

**Table 3:** Intrinsic motivation during the training in the ExerCube and the endurance training.

**Subscales**	**ECT**	**ET**	***P*** **-Values**
Interest/Enjoyment	13.9 ± 1.5	8.3 ± 2.4	<0.001
Perceived competence	11.6 ±1.5	10.7 ±2.2	0.065
Pressure/Tension	12.1 ± 2.3	13.0 ± 2.2	0.136
Total score^*^	37.6± 3.9	32.1 ± 5.4	<0.001

*ECT, ExerCube training; ET, endurance training*.

* *Total score is calculated from the presented subscales*.

Participants showed significantly smaller changes in pSBP, pDBP, cSBP, cDBP, HR, and pulse wave velocity after ECT compared to the corresponding parameters measured before ECT. The ET showed no significant differences in hemodynamic reactivity from pre- to post-exercise. The changes in hemodynamic reactivity were significantly higher for the ECT compared to the ET with respect to pSBP [*F*_(1,52)_ = 9.356, *p* = 0.004], pDBP [*F*_(1,52)_ = 7.354, *p* = 0.009], cSBP [*F*_(1,52)_ = 10.295, *p* = 0.002], cDBP [F_(1,52)_ = 7.056, *p* = 0.01], and pulse wave velocity [*F*_(1,52)_ = 5.091, *p* = 0.028; Table 4]. The participants reported a significantly [*F*_(1,52)_ = 8.281, *p* = 0.02] greater decrease in pain sensation after the ECT (−0.3) compared to ET (+0.6). Sex had no significant effect on hemodynamic parameters.

**Table 4:** Hemodynamic responses to the cold pressor test before and 45 min after training in the ExerCube and endurance training.

**Outcome**	**ECT**		**ET**		
	**CPTpre**	**CPTpost**	**CPTpre**	**CPTpost**	***P*** **-Values**
pSBP (mmHg)	14 ± 11.1	8 ± 9.0^**^	13 ± 9.1	15 ± 9.6	*P* = 0.004
pDBP (mmHg)	14 ± 8.8	12 ± 8.2^**^	13 ± 8.5	14 ± 9.9	*P* = 0.009
HR (m^−1^)	2 ± 5.6	−1 ± 4.3^***^	1 ± 8.6	1 ± 9.1	*P* = 0.104
cSBP (mmHg)	14 ± 10.3	5 ± 6.6^***^	14 ± 9.6	14 ± 11.6	*P* = 0.002
cDBP (mmHg)	14 ± 9.4	12 ± 8.2^*^	13 ± 9.4	14 ± 10.2	*P* = 0.010
PWV (m/s)	0.32 ± 0.39	0.04 ± 0.27^***^	0.36 ± 0.39	0.30 ± 0.36	*P* = 0.039

*ECT, ExerCube training; ET, endurance training; CPTpre, cold pressor test before exercise; CPTpost, cold pressor test 45 min after exercise; pSBP, peripheral systolic blood pressure; pDBP, peripheral diastolic blood pressure; cSBP, central systolic blood pressure; cDBP, central diastolic blood pressure; PWV, pulse wave velocity*.

* *p < 0.05*,

** *p < 0.01*,

*** *p < 0.001 changes from pre to post for ECT and ET. P-values represent interaction effects*.

## Discussion

The major and novel finding of the present study is that the ECT presents a high-intensity exercise stimulus that can reduce the hemodynamic reactivity to a stressor more than a conventional ET in healthy, young adults. Furthermore, the participants reported higher intrinsic motivation to participate in the ECT than in the ET.

According to the HRmean and VO_2_mean, the session in the ExerCube qualifies as a vigorous-intensity exercise (26). In terms of MET, the ECT can also be classified as a vigorous-intensity exercise for this specific age group (20–39 years). The exercise intensity was significantly higher than during a moderate conventional ET. Compared to other commercial exergames, the exercise intensity achieved during the ECT was considerably higher (5).

The relatively high exercise intensity that was achieved during the ECT can probably be attributed to the game setup and the innovative game design. As the ExerCube monitors the performance throughout the game and adjusts the challenge and speed of the game accordingly, an individually tailored training stimulus can be achieved.

Furthermore, the motion capturing system of the ExerCube monitors the precise execution of the movement tasks and thus can prevent cheating. Other gaming systems, especially those that use hand-held devices, may only require hand-movements to play the game. Often quick movements of the controller are sufficient to trick the motion capturing system and simulate large-scale movements.

Moreover, the whole-body movement tasks implemented in the game guarantee the engagement of large muscle groups. The relative amount of muscle mass activated during exergaming has been shown to be a key determinant of the exercise intensity attained during the game (27).

The hemodynamic results reveal that the ECT changed the reactivity to cold pressor test more than the ET. The peripheral and central BP responses stimulated by the cold pressor test were significantly lower after the ECT than before. In addition, the reduction in the respective parameters from pre- to post-training was significantly higher in the ECT compared to the ET.

The effect of acute physical exercise on stress-related hemodynamics has been studied previously. Ebbesen et al. (15) reported a dampening effect after 1 and 2 h of aerobic exercise (at 55% of VO_2_max) on cold pressor test-mediated increases in cardio-vascular variables in healthy adults. Similarly, Milatz et al. (17) were able to show lower hemodynamic responses after 60 min of moderate endurance exercise (at 45 % of VO_2_max). In the present study, however, the ET did not significantly reduce the stress response. Compared to the study by Ebbesen et al. (15), however, the participants in the present study had a higher fitness level. It could be argued that the exercise intensity of the ET was not high enough in magnitude to trigger relevant responses in this specific cohort. Furthermore, the exercise duration was considerably shorter compared to the study by Milatz et al. (17) and Ebbesen et al. (15). Thus, it can be speculated that there might be a dose-response relationship. This is supported by Saab et al. (28), who found no suppression of pSBP in response to the cold pressor test following 30 minutes of aerobic exercise (60 % of VO_2_max). On the contrary, Ebbesen et al. (15) found no differences between 1 and 2 h of aerobic exercise. Accordingly, exercise bouts over at least 35 min appear to be required to induce changes in hemodynamic reactivity.

However, it has been shown that shorter exercise sessions of higher intensities also reduce BP responses to the cold pressor test. In a previous study, high-intensity interval training consisting of six 1 min workouts at 98% of the maximum power significantly reduced the peripheral and central pressure response (16). This is consistent with the present results which show significant changes in hemodynamic reactivity after only 27 min of high-intensity and intermittent exergaming. This indicates that not only the duration of the exercise but also the intensity seems to have a modulating effect. In general, exercise intensity has been shown to be an important variable for training responses. Acute high-intensity exercise has been shown to produce more pronounced cardiovascular changes than moderate-intensity exercise (29).

On the contrary, Morissette et al. (30) were unable to show any changes in the cold pressor test-response after 30 seconds of an all-out exercise bout. It can therefore be hypothesized that both exercise intensity and duration appear to be relevant variables that modulate hemodynamic stress response. Further studies are warranted to determine possible intensity and duration thresholds.

When comparing the results with the findings from other studies, it must be pointed out that specific guidelines for conducting the cold pressor test are still lacking (13). Thus, test procedures can slightly vary between the different studies (temperature, duration). Furthermore, non-responders to the cold pressor test have been reported in previous studies (30), who could have been responsible for the inconsistent results.

Specific mechanisms for the suppressed cardiovascular response to the cold pressor test after exercise remain elusive, as the vascular responses to the cold pressor test are physiologically complex and not yet fully understood. However, as the cold pressor test stimulus includes a pain and cold component, it has been suggested that both may cause an increased cardiovascular response. Pain causes an increase in HR and BP by activating the sympathetic nervous system (31). Recent research indicates that acute anaerobic exercise can mitigate the sensation of pain (32), possibly decreasing the cold pressor test-mediated cardiovascular response following exercise. In the present study, the participants reported a significantly greater decrease in pain sensation after the ECT compared to ET. Furthermore, HR response during the cold pressor test after the ECT was lower compared to the cold pressor test before the exercise. Pickering and Gerin (33) assume that an increased activity of the sympathetic nervous system during the cold pressor test can result in significant α-adrenergic vasoconstriction with increased total peripheral resistance and a subsequent increase in BP. It is speculated that vasoconstriction due to post-exercise cold-exposure is mitigated, thereby reducing the cold pressor test-mediated cardiovascular response following exercise (15). This is supported by the present results which show a significantly lower HR, and pulse wave velocity after the ECT. Unfortunately, heart rate variability parameters have not been assessed.

The significant difference between the two exercise protocols may be attributed to the relative high exercise intensity during the ECT. This could have resulted in a higher oxygen demand provoking a greater blood flow through the vessel and, therefore promoted greater shear-stress induced nitric oxide (NO) distribution (34). Persistent shear stress-induced vasodilation that is not matched by an increase in cardiac output (35) is recognized as a cause of post-exercise hypotension and could also affect cardiovascular response during the cold pressor test. Another possible explanation of the different cardiovascular reactions between the two exercise protocols could be an exercise intensity dependent alteration in baroreceptor function with subsequent sympathetic inhibition (36). A higher fluid loss *via* sweat can also be discussed. However, the weight loss after ECT (−0.39 ± 0.27 kg) and ET (−0.30 ± 0.41 kg) was not significant.

Cardiovascular reactivity, defined as the magnitude of an individual's hemodynamic responses to a stressor, can be a marker for cardiovascular risk. According to Matthews et al. (37), BP hyperreactivity may reflect a more general hyperadrenergic state, with elevations in neurohormones leading to an increased risk for future hypertension. Furthermore, it may reflect endothelial dysfunction with an inability of the endothelium to adequately counteract the vasoconstrictive forces induced by sympathetic stimuli (37). Cardiovascular reactivity is not only a marker of cardiovascular disease, but it can also be a mechanism in the pathogenesis of cardiovascular diseases. It has been suggested that frequent fluctuations in sympathetic activity and thus changes in blood pressure can have direct effects on the vascular system, causing damage and affecting arterial compliance which in the long term can result in hypertension (38). This is of relevance as both chronic and acute stress is an inherent element of everyday life.

Various studies have shown that cardiovascular reactivity can predict future cardiovascular morbidity and mortality (12, 14). Thereafter, attenuating cardiovascular responses to stress can play an important role in preventing cardiovascular diseases. According to the literature, this response can be modulated through regular and acute exercise (15, 16).

With respect to the present results, even an acute exergaming session may induce these positive effects. However, it seems that the exercise stimulus must attain a certain intensity and/or duration threshold to induce relevant physiological adaptions. Accordingly, most current exergames, which only trigger low to moderate intensity (5) may not trigger these responses. Nevertheless, it must be considered that the participants in the current study had a relatively high fitness level. It could be argued, that less intensive exergames may induce a relevant exercise stimulus in inactive individuals or risk patients.

Unfortunately, no previous studies have assessed the effects of an exergaming session on stress test-related hemodynamics. Nevertheless, a few studies were able to show effects of acute exergaming on different hemodynamic parameters at rest (39–42) supporting our findings.

Apart from triggering a physiologically relevant exercise stimulus, the intrinsic motivation during the ECT was rated significantly higher than during the ET, suggesting that the participants were intrinsically motivated to participate in the ECT. This is of relevance as intrinsic motivation has been reported to be one of the strongest determinants of exercise adherence and is highly associated with enjoyment (43). Intrinsic motivation refers to engaging in an activity purely for the pleasure and satisfaction derived from doing the activity (43). Thereafter, the results support the use of the ExerCube as an attractive and motivating tool to promote physical activity. The higher perceived intrinsic motivation, and the significantly higher scores in the subscale interest and enjoyment during the ECT might be attributed to the immersive and distracting experience provided by the audio-visual game scenario and the tailored training stimulus. However, the ECT could have benefited from a novelty effect as none of the participants had any experience with the ExerCube prior to this study. Soltani et al. (44) determined greater levels of enjoyment in inexperienced compared to experienced exergame players. Furthermore, the ECT was compared with treadmill jogging under laboratory conditions. The enjoyment and flow experience of treadmill running may differ from real-life conditions.

To date, the ExerCube is one of the few exergames that enable an individually tailored high-intensity training program. Irrespective of the high training intensity exercising in the ExerCube presents a joyful training stimulus. Furthermore, it is the first exergame shown to affect hemodynamic reactivity. Thus, this exergame may present a promising training tool for different target groups and settings. In particular, the innovative approach of continuously adjusting the game challenge and speed to the performance and the possibility to set a HR threshold can make it suitable for cardiovascular rehabilitation and even risk patients. However, as the ExerCube requires a relatively large space (9 m^2^), it may not be practical for home-based training. It rather represents a training tool for gyms, prevention, or rehabilitation centers. Due to the cross-sectional study design, it is not clear whether the higher enjoyment possibly affects long-term exercise adherence and if the acute physiological effects could accumulate.

Nevertheless, it can be assumed that properly designed exergames may present an effective alternative to traditional training approaches and can help expand cardiovascular prevention approaches. However, further longitudinal studies in different populations and settings are highly warranted.

## Limitations

There are a few methodological limitations that warrant discussion. First, only young, healthy white individuals with a relatively high fitness level were included. Research shows that cardiovascular reactivity can differ in different populations and ethical groups (14). However, according to previous researchers, blood pressure response during the cold pressor test is not affected by fitness level (46). Nevertheless, further studies including different populations, especially risk patients, should be carried out. As the physiological mechanisms for the hemodynamic responses to the cold pressor test have not been fully elucidated it is not clear if this study controlled for all relevant confounding factors that contribute to the physiological response. Thus, future studies are warranted assessing possible factors that could bias the results.

Second, a stepwise incremental test was used to determine HRmax and VO_2_max. A ramp-wise incremental graded exercise test might have been superior for determining maximum values. However, the stepwise test was used to ensure a steady state for the lactate assessment.

Third, the results are limited to the game setting (ExerCube) and the exercise protocol (sphery racer) applied in the present study. Other exergames will likely produce different effects.

Fourth, there was a significant difference in the exercise intensity between the two exercise protocols. As the exercise intensity during the ECT was not clear before the study a very general exercise protocol that has been widely proven to modulate cardiovascular responses to the cold pressor test (15, 17) was chosen. Future studies should compare cardiovascular and hemodynamic responses between the ECT and exercise protocols of similar intensities.

Fifth, heart rate variability parameters were not included in this study. Assessing changes in autonomic function could have helped to analyze the results better.

Finally, the effects were only examined after an acute ECT. Conclusions on long-term results are not possible. It, therefore, remains questionable if the ECT can provide an effective and engaging training environment over longer periods of time. Especially the lack of social interaction can decrease enjoyment over time. Further studies assessing the long-term effects of ECT are highly warranted.

## Conclusion

In conclusion, the present findings are the first to illustrate the effects of exergaming on cardiovascular reactivity to the cold pressor test. The results show that the ECT is a vigorous exercise stimulus that can attenuate the hemodynamic reactivity to a cold pressor test and results in high levels of enjoyment in young, healthy adults. Due to its individually tailored training approach, the ECT can present a promising tool for the prevention of cardiovascular diseases and may even be applied in rehabilitation settings.

## Data Availability Statement

The raw data supporting the conclusions of this article will be made available by the authors, without undue reservation.

## Ethics Statement

The studies involving human participants were reviewed and approved by Institutional Research Committee of the Medical Faculty of the Martin-Luther-University Halle-Wittenberg. The patients/participants provided their written informed consent to participate in this study.

## Author Contributions

SK, KK, and RK conceived the original idea and designed the study. SK drafted the manuscript and designed the figures. KK and KH supervised the project. LR and EK performed the measurements. AM-N design the exergame intervention and gave technical support. EK and SK processed the experimental data and performed the analysis. KK, RK, EK, AM-N, and KH aided in interpreting the results and worked on the manuscript. All authors provided critical feedback and helped shape the research, analysis, and manuscript. All authors have read and approved the final version of the manuscript.

## Funding

We acknowledge the financial support from the funding program Open Access Publishing by the Martin-Luther-University Halle-Wittenberg.

## Conflict of Interest

Besides being a Senior Researcher at the Zurich University of the Arts, AM-N is also Co-Founder and CEO of the spinoff company Sphery. No revenue was paid (or promised to be paid) to AM-N, to Sphery or the research institutions. The remaining authors declare that the research was conducted in the absence of any commercial or financial relationships that could be construed as a potential conflict of interest.

## Publisher's Note

All claims expressed in this article are solely those of the authors and do not necessarily represent those of their affiliated organizations, or those of the publisher, the editors and the reviewers. Any product that may be evaluated in this article, or claim that may be made by its manufacturer, is not guaranteed or endorsed by the publisher.
